# 136 Link workers promoting physical activity - who are they linking?

**DOI:** 10.1093/eurpub/ckae114.016

**Published:** 2024-09-26

**Authors:** Megan O’Grady, Emer Barrett, Deirdre Connolly

**Affiliations:** Discipline of Physiotherapy, Trinity College Dublin, Ireland; Discipline of Physiotherapy, Trinity College Dublin, Ireland; Discipline of Occupational Therapy, Trinity College Dublin, Ireland

## Abstract

**Purpose:**

Improvements in health and wellbeing can be achieved in individuals who are connected to community and voluntary services though link workers (LW). There is emerging evidence of their potential role in promoting local, community-based physical activity (local PA). More research is needed to understand the characteristics of individuals who attend this intervention, including health and demographic information.

**Methods:**

This abstract presents results from two studies. In Study 1, semi-structured interviews were conducted with 27 Irish LW, including local sports partnership officers (LSPO) and social prescribing link workers (SPLW), to describe the profile of their service users. Study 2 (currently ongoing) is a mixed-methods pilot feasibility study. Demographic, PA (International Physical Activity Questionnaire - Short Form, Self-Efficacy for Exercise Scale) and health-related data (Short Warwick Edinburgh Mental Well-being Scale) were collected from 23 individuals attending a LW intervention, which included referral to local PA.

**Results:**

In Study 1, Irish LW reported that their service users were often mid- to older-aged adults with significant physical and mental health comorbidities (including social isolation) which limited participation in PA. Link workers addressed these barriers to PA throughout their intervention. In Study 2, fifteen participants were recruited from LSPO and eight from SPLW. Participants were predominantly female (20/23, 87%) with a mean age of 69.2 (SD 6.7). Six in ten (14/23, 61%) reported a chronic disease. Almost half did not meet current PA guidelines (11/23, 48%) and had moderate levels of self-efficacy for exercise (mean 54.8 (SD 16.1)). Six in ten (14/23, 61%) reported only moderate levels of wellbeing. Preliminary analysis of PA and health-related data shows differences between LSPO and SPLW groups.

**Conclusions:**

Irish LW service users experience many barriers to PA including social isolation, chronic disease and reduced self-efficacy for exercise. They may benefit from individualised support and targeted referrals to local PA provided by LW. By examining the potential differences in characteristics of people attending SPLW and LSPO services we hope to inform future referral pathways.

**Support/Funding Source:**

Supported by the Glennon Bursary, awarded by the Irish Society of Chartered Physiotherapists: January 2023 – January 2024.

